# Marble Algorithm: a solution to estimating ecological niches from presence-only records

**DOI:** 10.1038/srep14232

**Published:** 2015-09-21

**Authors:** Huijie Qiao, Congtian Lin, Zhigang Jiang, Liqiang Ji

**Affiliations:** 1 Key Laboratory of Animal Ecology and Conservation Biology, Institute of Zoology, Chinese Academy of Sciences, Beijing 100101, China

## Abstract

We describe an algorithm that helps to predict potential distributional areas for species using presence-only records. The Marble Algorithm is a density-based clustering program based on Hutchinson’s concept of ecological niches as multidimensional hypervolumes in environmental space. The algorithm characterizes this niche space using the density-based spatial clustering of applications with noise (DBSCAN) algorithm. When MA is provided with a set of occurrence points in environmental space, the algorithm determines two parameters that allow the points to be grouped into several clusters. These clusters are used as reference sets describing the ecological niche, which can then be mapped onto geographic space and used as the potential distribution of the species. We used both virtual species and ten empirical datasets to compare MA with other distribution-modeling tools, including Bioclimate Analysis and Prediction System, Environmental Niche Factor Analysis, the Genetic Algorithm for Rule-set Production, Maximum Entropy Modeling, Artificial Neural Networks, Climate Space Models, Classification Tree Analysis, Generalised Additive Models, Generalised Boosted Models, Generalised Linear Models, Multivariate Adaptive Regression Splines and Random Forests. Results indicate that MA predicts potential distributional areas with high accuracy, moderate robustness, and above-average transferability on all datasets, particularly when dealing with small numbers of occurrences.

Finding accurate ways to estimate species’ ecological niches is a pressing and important problem in ecology and biogeography^1^,^2^,^3^. Consequently, a wide variety of modeling techniques have been developed for this express purpose^4^,^5^,^6^. Most such models, often referred to as ecological niche models (ENM), use the correlation between environmental factors and the distribution of a species to predict the potential distributional area within a wider unknown space (be it geographic or environmental)^6^,^7^. Popular modeling choices include Bioclimate Analysis and Prediction System (BIOCLIM)^8^, the Genetic Algorithm for Rule-set Production (GARP)^7^, Generalised Boosted Models (GBM)^9^, Generalised Linear Models (GLM)^10^, and Maximum Entropy Modeling (MaxEnt)^11^,^12^,^13^, among others. The mathematical equations underlying these modeling programs often vary. For example, the core algorithm underpinning GARP is one of machine learning, whereas MaxEnt uses the probability theory principle of maximum entropy. ENMs are widely used in predicting the loss of species arising from global climate change^14^,^15^,^16^, planning protected areas^17^, calculating public health problems caused by the spread of disease^18^,^19^, comparing the niche breath on species survival with fossil records^20^, and modeling the spread of invasive species^21^,^22^,^23^.

In this paper, we present a cluster-based ENM algorithm, the Marble Algorithm (MA), which represents an integrated spatial analysis program for estimating the ecological niche of a given species. MA utilizes the density-based clustering algorithm DBSCAN^24^, which searches for multi-clusters in environmental space and maps these clusters onto geographic space. MA is developed as a plug-in of a web-based application, mMWeb (http://mmweb.animal.net.cn)^25^,^26^, which combines 20 currently-existing ENM algorithms. Users can employ MA via any browser supported by mMWeb.

We compared the performance of MA with 12 widely-used ENM algorithms, including BIOCLIM, Environmental Niche Factor Analysis (ENFA), GARP, MaxEnt, Artificial Neural Network (ANN)^27^, Climate Space Model (CSMBS)^28^, Classification Tree Analysis (CTA)^29^, Generalised Additive Models (GAM), GBM, GLM, Multivariate Adaptive Regression Splines (MARS)^30^, and Random Forests (RF)^31^,^32^, using both virtual species and empirical data sets. Four metrics were used to compare the resulting ENMs, including the true skill statistic (TSS), Cohen’s kappa, partial-area ROC (P-ROC), and transferability. Climate layers used in the experiments were downloaded from WorldClim (http://www.worldclim.org/).

## Results

### Model performance on virtual species

**Figure 1:**
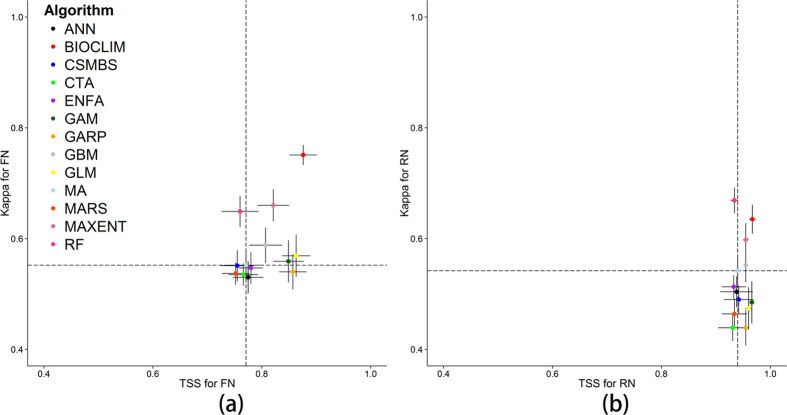
Average performance of the 13 ENM algorithms in predicting the FN (**a**) and RN (**b**), as measured via Cohen’s kappa and True Skill Statistic (TSS) (average ± 95% confidence error). The dash lines represent the TSS and Kappa value of MA.

MA had average performance in predicting both the FN and RN compared to other ENM algorithms (Fig. 1). When estimating the FN, eight (BIOCLIM, GAM, GARP, GBM, GLM, MAXENT, ENFA and ANN) and six (BIOCLIM, GAM, GBM, GLM, MAXENT and RF) of the 13 algorithms performed better than MA when assessed using the TSS and Cohen’s kappa metric, respectively (Fig. 1a). MA performance improved when estimating the RN. Seven (BIOCLIM, GAM, GARP, GBM, GLM, MAXENT and CSMBS) and four (BIOCLIM, GBM, MAXENT and RF) models performed better than MA when estimating the RN using the TSS and Cohen’s kappa metric, respectively (Fig. 1b).

### Model accuracy

**Figure 2:**
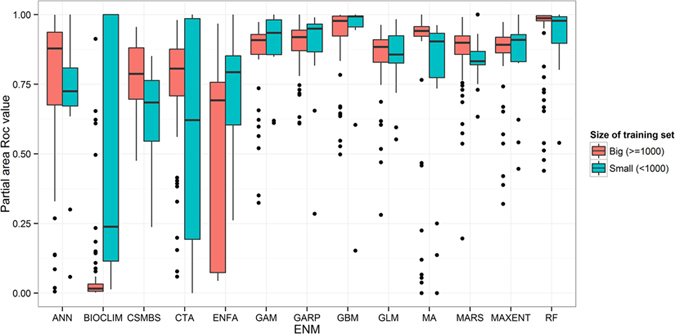
The partial-area ROC values of the 13 models utilizing different training set sizes, which are shown by the boxplots. The red boxplots are partial-area ROC values for the large training sets, and the green boxplots represent the small training sets.

Eight models (GLM, GARP, MARS, GAM, MAXENT, GBM and RF), including MA, predicted the FN and RN with high accuracy based on the mean value of the P-ROC indicator using both the large and small occurrence datasets (Fig. 2). Three models (MAXENT, GBM and RF) performed better than MA using the large occurrence datasets, and seven models (GLM, MARS, GARP, MAXENT, GAM, GBM, and RF) performed better than MA using the small occurrence datasets. Since the mean P-ROC values for the top eight models, which includes MA, were all higher than 0.8 (i.e., excellent), it was difficult to draw conclusions on the performance of the models based solely on the P-ROC values. That is to say, we could not ascertain whether one model was better than another on a case-by-case basis: only that a model was better than another based on overall average performance.

**Figure 3:**
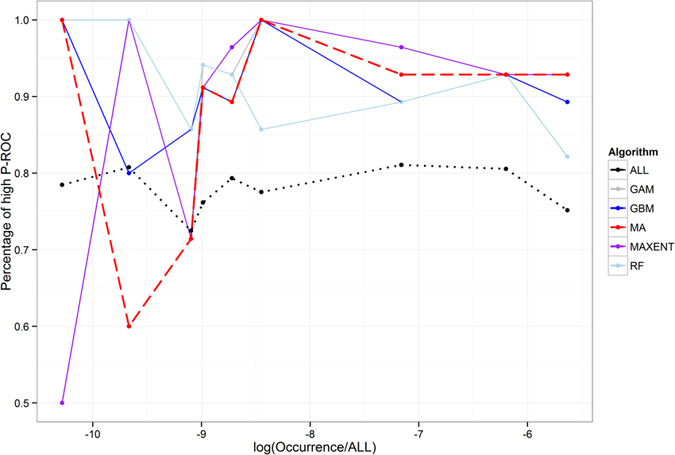
Percentage of partial-area ROC (P-ROC) analyses above 0.8 for the different datasets. The x-axis represents the log of the rate of occurrences, which is used to represent the relative sample sizes. The y-axis is the percentage of P-ROC values that are >0.8. The black dashed line is the average P-ROC value for all the algorithms, and the red dashed line is the average P-ROC value for MA. All algorithms with average P-ROC values higher than MA are shown with solid lines in different colors.

Therefore, in order to characterize the performance and accuracy of the models more precisely, we calculated the rate of excellent P-ROC values (i.e., >0.8) divided by the ratio of the number of occurrence localities and the number of grid cells in the study area as a whole. Since the number of occurrence records was far less than the number of grids in the entire study area for most datasets, we used a Semi-log plot to bring out features in the data not easily observed if they were plotted linearly. The percentage of high P-ROC values for MA was higher than the mean value of all models when considering the large ratios of the number of occurrence localities and the number of grid cells (see Fig. 3).

### Transferability indicator

**Figure 4:**
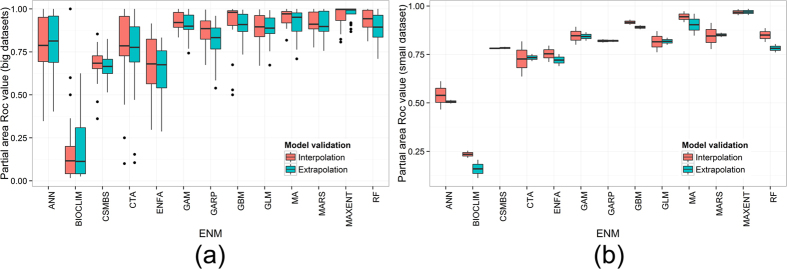
Mean accuracy of the 13 models for interpolative and extrapolative validation: (**a**) large datasets split by an 8*8 checkboard. (**b**) small datasets split by an 8*8 checkboard.

**Figure 5:**
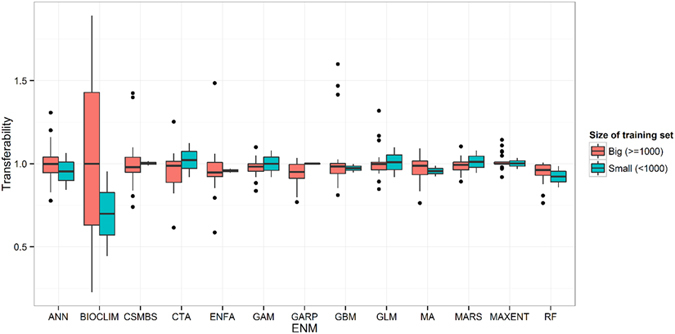
Model transferability of the 13 models. Transferability is calculated as the ratio of P-ROC value in extrapolative *versus* interpolative model validation for each group separately.

On average, MA received relatively high interpolation and extrapolation P-ROC values using both the large and small occurrence datasets (Fig. 4). Based on these interpolation and extrapolation indicators, Maxent performed best when considering both the large and small occurrence datasets. Following Maxent, MA and GBM received the highest scores, although GBM was more stable than MA based on the boxplots. MA did not perform as well compared to other models when considering the transferability indicator for both the large and small occurrence datasets (Fig. 5).

## Discussion

### Accuracy and scope of applicability

Successful ENMs should have reasonable accuracy when predicting potential distributional areas. In this regard, results from both virtual and real species indicated MA produces results with higher-than-average accuracy in most test cases. Although some models produced results with higher accuracy than MA in some datasets, this may be a function of over-fitting in those algorithms. That is to say, the model produced a potential distribution that was very similar to the training set (i.e., the input occurrences). In the process of modeling, the performance may increase for training examples, but may decrease when considering unseen data. In most cases when niche modeling, a balance is desired between high accuracy on the training set and scalability. MA tends to predict large areas as suitable for a given species with relatively high accuracy, which illustrates the ability of MA to prevent overfitting even when training on datasets with few occurrences.

The ratio of number of occurrences and number of grid cells in the entire study area, which is sometimes referred to as a movement region^33^, seriously affects model validation and model comparisons. Consequently, obtaining sufficient data and choosing a suitable study area should be prime considerations of any ENM analysis, no matter the algorithm employed.

### Extrapolation capabilities and transferability

The extrapolative value and transferability index are new evaluation indicators, and ecological niche modeling algorithms should be tested using these new metrics. Based on the P-ROC results, MA performed second only to Maxent for extrapolation capability and had average performance for transferability when the study areas were relatively small. However, MA had below average performance for both extrapolation capability and transferability when the study areas were large.

## Conclusions

We describe a new model based on the DBSCAN algorithm for predicting species’ potential distributional areas with only positive-presence examples. MA builds a model to estimate the potential distributional area for species based on occurrence localities and environmental factors that affect the distribution of species. Similar to GARP and MaxEnt, MA accounts for differences among populations of the same species and is designed to divide the occurrence samples into several groups and cluster them separately. MA can handle the correlation between environmental factors and eliminates a small amount of outliers (noise) before modeling. When modeling with a high-dimension sample set, and/or with large or small numbers of occurrences in a small study area, MA is a recommended model.

MA, however, still has shortcomings when compared to MaxEnt and RT. Results were suboptimal when working with a small data set on a large study area, for which the number of grid cells of the study area is 10^6^ times the number of occurrence points. Moreover, only continuous data can be used to calculate distance in MA. Finally, MA produces a presence (0) or absence (1) map, which is not an output desired by many users (i.e., instead preferring a continuous map of suitability). We hope to solve these problems in future updates.

## Methods

MA is based on the density-based clustering algorithm DBSCAN^24^. The algorithm assumes that the sample sets of *X* are concentrated in some dense space of the set *S*, which is named as a cluster. The algorithm searches for clusters of species’ occurrences in environmental space, which will produce a niche map resembling water insoluble marbles found in clay rocks. In MA, the definition of a cluster (the distribution of a population) is based on the notion of density reachability, and follows the original definitions of DBSCAN. The concept of “distance” in MA is not geographic, but rather ecological or environmental (i.e., ecological niche distance).

### Details of the MA algorithm

#### Definitions

The algorithm’s main process is to find clusters according to given rules:

##### Definition 1

Point (*p*) is a set of environmental variables associated with an occurrence in the sample set. *Eps* is the distance between two points. *MinPts* is the minimum number of points required to form a cluster.

##### Definition 2

*Eps*-neighborhood of *p.* For a given point *p*, the distance between *p* and any point *q* in a set *N*_*Eps*_*(P)* in the vicinity of *Eps*. The set of *N*_*Eps*_*(P)* denotes the *Eps*-neighborhood of *p*. The “distance” denotes the niche disparity of these two localities:


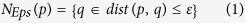


##### Definition 3

**Figure 6:**
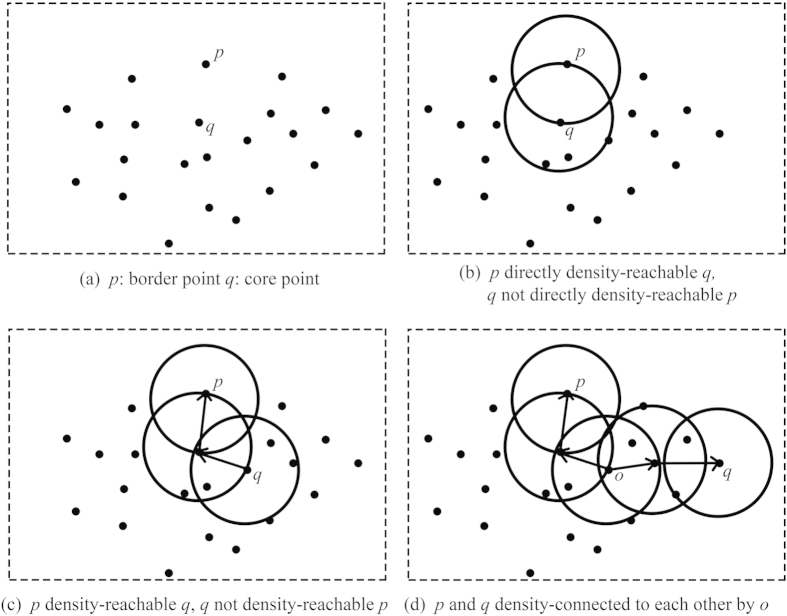
The basic concepts used in the Marble Algorithm, which derive from DBSCAN.

Core, border and noise points. For a given point *q*, if there are at least a minimum number (*MinPts*) of points in the *Eps*-neighborhood of *q*, the point *q* is defined as a core point. If the number of points in the *Eps*-neighborhood of point *p*, which is one of the *Eps*-neighborhood of *q*, is less than *MinPts*, the point *q* is defined as a border point (Fig. 6a). If a point *n* is neither a core point nor a border point of any core point, it is defined as a noise point.

##### Definition 4

Directly density-reachable. For the given *Eps* and *MinPts*, a point *p* is directly density-reachable from a point *q* should it satisfy the following conditions (Fig. 6b):


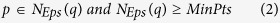


##### Definition 5

Density-reachable. If there is a chain of points *p*_*1*_*,…, p*_*n*_*, p*_*1*_ = *q, p*_*n*_ = *p,* such that *p*_*i*_ _*+*_ _*1*_ is directly density-reachable from *p*_*i*_, we call the point *p* density-reachable *q* (Fig. 6c).

##### Definition 6

Density-connected. If the points *p* and *q* are not density-reachable, but both are density-reachable from point *o*, we call *p* and *q* density-connected by *o* (Fig. 6d).

##### Definition 7

Cluster (population). Let *D* be a dataset of points. A cluster *C* wrt. *Eps* and *MinPts* is a non-empty subset of the set *D* satisfying the following conditions: (1) there is at least a core point; and (2) each point in *C* should at least be density-connected to a core point in *C*. A population of a given species is regarded as a cluster in MA. All the organisms in a population have some relationship (density-connected). A given dataset has one or more clusters (different populations).

##### Definition 8

Noise. Let *C*_*1*_*,…, C*_*k*_ be the clusters of the dataset *D* wrt. parameters *Eps* and *MinPts*. The noise is defined as the set of points in the dataset *D* not belonging to any cluster *C*_*i*_, _*i*_ _*=*_ _*1,…,k*_.

### Algorithm implementation

Based on the above definitions, estimating the ecological niche becomes a process of searching for clusters in a given dataset. After identifying clusters, all points in the unpredicted area will be added to the dataset in turn. If a point belongs to a cluster, it is considered within the potential distribution of the given species, or *vice versa*.

To find a cluster of given *Eps* and *MinPts*, MA starts with an arbitrary point *p* in an unclassified set *D* and retrieves all points density-reachable from *p*. (1) If *p* is a core point and *p* is not labeled, a new cluster *C*_*i*_ will be built. The point *p* and the points of density-reachable *p* will be labeled as the points in *C*_*i*_. Subsequently, the algorithm will randomly select an untreated point in *C*_*i*_ and run the process above recursively. (2) If *p* is not a core point, it will be temporarily marked as noise unless it is selected as a core point later.

**Figure 7:**
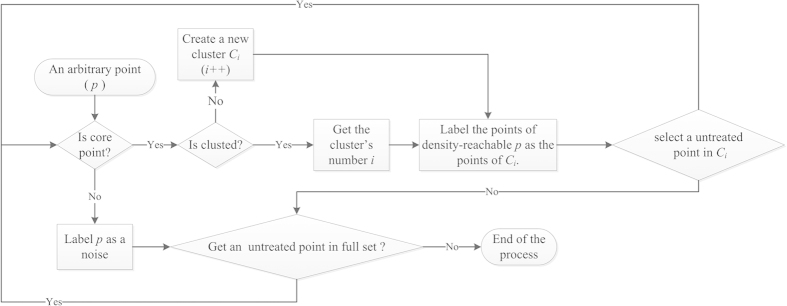
Flow chart for MA.

After labeling all the related points to cluster *C*_*i*_, the algorithm picks another random unclassified point in *D* and repeat the steps above until each point in *D* has been labeled (Fig. 7).

### Distance

For the purpose of proper visualization, we use the Euclidean distance in 2D space to describe the algorithm. We assume that *D* is the niche disparity of two occurrence localities in n-dimensional environmental space. *E*_*i*_ = (*e*_*1*_*, e*_*2*_*, …, e*_*n*_) are the ecological factors (biotic and abiotic factors) at location *i*. Therefore, the distance of *i* and *i* + *1* is:


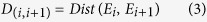


As we only observe portions of the ecological factors 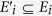, a distance *D’* is used to approximate *D*. It is denoted as: 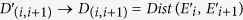


Euclidean distance is the spatial distance between two points in the Cartesian coordinate system. Coordinate axes are measured in the same unit of length and are orthogonal to each other. The problem, however, is that each ecological factor may have a different dimension (such as Meter, Celsius, etc.) and may exhibit correlation with other factors. MA provides a solution to these problems, however, by normalizing the factors and performing principal component analysis (PCA) instead of using the Euclidean distance directly:

PCA transforms a number of possibly-correlated variables into a number of uncorrelated variables called principal components. Before PCA, MA converts the values of each variant to a standard score. Equation (4) illustrates the conversion method, where x is a raw score to be standardized, μ is the mean of the variant, and σ is the standard deviation of the variant.


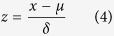


After normalization, MA calculates the principal components of the given variants and selects the first *n* principal components on the condition that the sum of the eigenvalues is no less than 0.95.

Since there is no correlation between two principal components, Euclidean distance is used to express niche disparity directly, and the dimensionality of the transformed data is thus reduced. Both PCA and normalization reduce the complexity the environmental space effectively, and make the algorithm clear and easy to understand.

### Determining parameters

*Eps* and *MinPts* are two necessary parameters of MA. *Eps* determines the area of the cluster. *MinPts* is the minimum number of records in a cluster. Both parameters impact the results of the model. The potential distribution of a species is expected to be the smallest area that contains all occurrence localities.

We developed a simple but effective heuristic to determine the parameters *Eps* and *MinPts* of the “smallest” clusters in the dataset. First, let *d*_*i*_ be the minimum distance between point *p*_*i*_ and all the other points. Then, build a set *D* such that 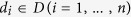. Afterwards, we obtain the maximum value *d*_*max*_ in *D.*

If we set *Eps* to *d*_*max,*_and *MinPts* to 2, then all the points are included in a cluster. However, if the cluster is not the excepted smallest result, the algorithm will increase the value of *MinPts* and execute the clustering process until some of the points are labeled as noise points. The penultimate value of *MinPts* in the iterative process is the recommended value.

**Figure 8:**
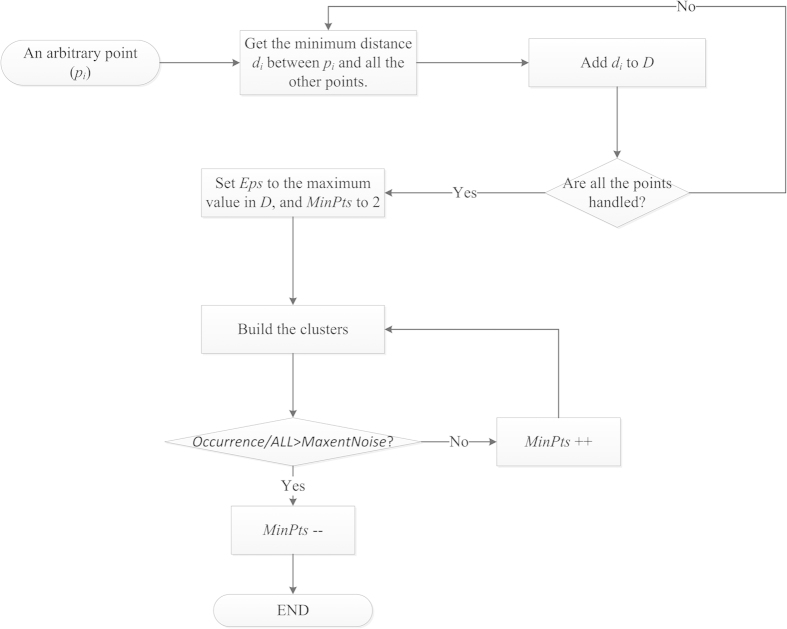
Flow chart for the process of determining the parameters.

Figure. 8 illustrates the process of determining *Eps* and *MinPts*. In order to increase the model’s flexibility, the parameter of *MaxNoise* is the maximum proportion of noise points in the samples, which is set by user based on his confidence in the occurrences used in MA. It means no noise in occurrences when *MaxNoise* is zero, and all the occurrences are noises when *MaxNoise* is 1.The concept of noise was interpreted in the next section.

### Handling noise

Occurrence samples are rare, especially for endangered species. Therefore, a small number of noise points in a dataset with few occurrences can have a strong impact on the results. Consequently, an independent method is introduced into MA to hunt down and remove noise data before the main algorithm process. Assume there is one noise point *p*_*i*_ in a dataset *S*, *D*_*i*_ is the set of distances between *p*_*i*_ and the other points, and *s*_*i*_ is the standard deviation of *D*_*i*_. Then, there should be the minimum value *s*_*n*_ in *S* given that *p*_*n*_ is the only noise point, since all values in *D*_*n*_ are large and there is only one large value beside *D*_*n*_.

MA does not use the minimum value but a more robust method, termed the interquartile range, to find noise points based on *S*^34^. The standard deviations of the distances between noise points and none-noise occurrences are observations that fall below a given value, which is called the “outlier factor” in MA. The outlier factor can be 1.346 (at a 95% confidence interval) or 1.469 (at a 99% confidence interval), which is decided by the user as a parameter in MA.

Identified noise points may actually represent real occurrences in unique environmental conditions. Under these conditions, it would be irresponsible for the algorithm to cursorily remove these data points. As such, MA provides a switch to turn off this feature if the user guarantees the accuracy of his/her dataset.

### Model comparison

#### Environmental variables

We used monthly temperature and rainfall layers downloaded from WorldClim^35^ to build the models. More specifically, we performed a principal component analysis on a group of normalized 19 bioclimatic variables, and selected the first six principal components that explained 95.7% of the overall variance. The grids of derived variables can be downloaded via http://mmweb.animal.net.cn/varlist.html.

### Datasets

#### Virtual species

Simulated species’ distribution data with known properties (termed virtual species) is commonly used to evaluate the performance of ENM without the confounding effects of data characteristics^36^,^37^,^38^. At present, at least four methods are available to generate virtual species^39^,^40^,^41^,^42^. In this paper, we created 14 virtual species following methods in a previous study^40^. The virtual species were generated via ellipsoids in a three-dimensional environmental space; these species were created with or without barriers to dispersal in a corresponding geographic space. Further details on the virtual species are presented elsewhere^40^.

#### Empirical datasets

In addition to the virtual species, we used ten empirical datasets to evaluate MA, including four mammal species (*Lynx lynx* Linnaeus, 1758 - Eurasian lynx, *Procapra przewalskii* Büchner, 1891 - Przewalski’s gazelle, *Oryctolagus cuniculus* Linnaeus, 1758 - European rabbit and *Cervus elaphus* Linnaeus, 1758 - red deer), one amphibian family (Cryptobranchidae), one insect species (*Apis mellifera* Linnaeus, 1758 - western honey bee), one bird species (*Syrmaticus reevesii* J. E. Gray, 1829 - Reeves’s pheasant), two vascular plant genera (*Mikania* and *Liriodendron*), and one vascular plant species (*Pinus sylvestris* Linnaeus*—*Scots Pine).

The datasets were divided into two groups based on numbers of occurrence records. The small sample group included the Eurasian lynx, Cryptobranchidae, *Mikania*, Przewalski’s gazelle, and the Reeves, and all contained fewer than 1000 occurrences. These two groups were used to compare the accuracy of MA and the other ENM algorithms based on the different dataset sizes. For the small-sample group, we predicted the potential distribution directly, whereas for the large-sample group, we selected 80% of the samples randomly as the training set and used the remainder as a testing set. The training set was used to construct the model and to test its accuracy. After ten repetitions, we regarded the average accuracy as the final result.

**Figure 9:**
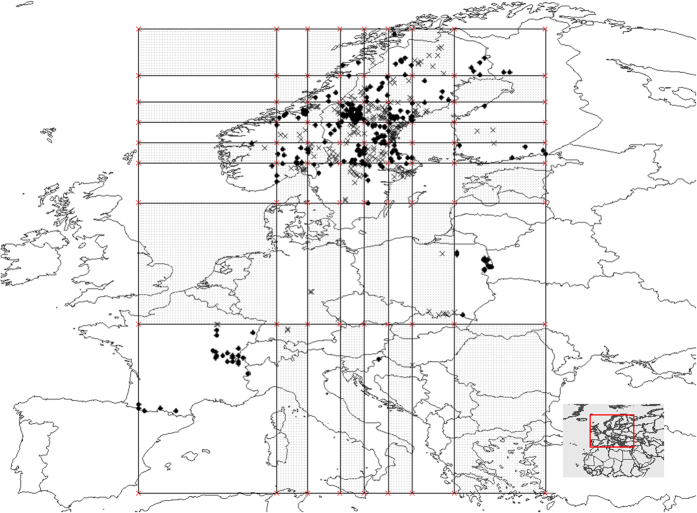
One of the study datasets (Eurasian lynx in Europe), which is divided into two spatially independent portions by a density-based checkerboard: “white background” or “grid background”. If the ‘extrapolation’ dataset is located on the white background, the grid background contains both ‘interpolation’ and ‘calibration’ datasets, and vice versa. This figure and the following figures are generated based on Thematic Mapping (http://thematicmapping.org/) using ArcGIS 9.2 (ESRI, Redland, CA).

In order to evaluate the interpolative and extrapolative model accuracy, datasets were subdivided using an 8 * 8 checkerboard (see Fig. 9 for an example). Given that the distribution of occurrences within each dataset was uneven, we split longitude and latitude into 8 parts based on the frequency of occurrences rather than on spatial distance. This process created a density-based checkerboard (Fig. 9), where grid area relates to the density of the samples inside. The method built relatively uniform sample subsets and broke up environmental trends over broad areas. Prior to model-building, each dataset was randomly split into two spatially-mixed datasets: a training dataset, including 70% of the samples, and an interpolation testing dataset, containing the remaining 30% of samples. In summary, the datasets were generated for different usages: (1) a training dataset (used to build the model), (2) the interpolation testing dataset (used to evaluate the interpolative model accuracy), and (3) the extrapolative validation dataset (used to evaluate the extrapolative model accuracy).

#### Modelling techniques

We used 12 ENM methods to build the models using default parameters: ANN, BIOCLIM, CSMBS, CTA, ENFA, GAM, GARP, GBM, GLM, MARS, MaxEnt, and RF. BIOCLIM, CSMBS, ENFA and GARP were implemented within openModeller, an open source project, for the entire process of conducting a fundamental niche modeling experiment^43^. MaxEnt (V3.3.3 k) was downloaded from http://www.cs.princeton.edu/~schapire/maxent/, while all other algorithms were implemented in BIOMOD^44^. To enable a standardized evaluation of all models, all predictions were made via mMWeb with the default or suggested parameters, including the method for selecting pseudo-absences. All results can be found on the platform (http://mmweb.animal.net.cn).

#### Evaluation indicators

To summarize the results, we took advantage of the “known-truth” nature of virtual species. In other words, the true geographic extent of the potential distribution was known (i.e., the fundamental niche, FN), as was the intersection of the potential distribution with the dispersal capabilities of the species (i.e., the realized niche, RN). To assess correspondence between model outputs and the known true configurations, we calculated Cohen’s kappa and TSS with respect to both FN and RN.

For the empirical datasets, we evaluated the predictive performance of the models using P-ROC metrics. This approach provides a firmer foundation for evaluating predictions from ecological niche models, which was noted previously by Phillips and Peterson^45^,^46^. For each model, a threshold for conversion of the continuous probability values for occurrence into binary predictions to distinguish ‘suitable’ (classified as 1) from ‘unsuitable’ (classified as a 0) areas was selected according to the lowest presence threshold. This threshold method identifies the minimum predicted area possible whilst maintaining zero omission error in the training data set^46^,^47^. The P-ROC values were interpreted in line with Swets^48^; that is, >0.80: excellent, 0.80–0.70: good, 0.60–0.70: fair, 0.50–0.60: poor and <0.50: fail.

To evaluate model transferability, the concept of the “transferability index” is introduced, which was discussed in Heikkinen *et al.*^49^. Based on the subsamples separated by the density-based 8*8 checkerboard, we calculated the interpolative and extrapolative P-ROC values, and subsequently obtained the transferability index for every training set. This index is equal to the ratio of the P-ROC value in the extrapolative versus the interpolative model validation and is performed for each dataset separately.

## Additional Information

**How to cite this article**: Qiao, H. *et al.* Marble Algorithm: a solution to estimating ecological niches from presence-only records. *Sci. Rep.* **5**, 14232; doi: 10.1038/srep14232 (2015).
